# Enterohemorrhagic *Escherichia coli* targets Annexin A6 and ATG16L1 to inhibit autophagy and induce inflammation

**DOI:** 10.3934/microbiol.2025044

**Published:** 2025-12-17

**Authors:** Litai Xu, Min Gao, Yaoguo Wang, Bao Zhang, Wei Zhao, Weizhi Lu, Guanhua Cao, Chengsong Wan, Ying Hua

**Affiliations:** 1 Department of Microbiology; BSL-3 Laboratory (Guangdong), Guangdong Provincial Key Laboratory of Tropical Disease Research, School of Public Health, Southern Medical University, Guangzhou, China; 2 Department of Laboratory Medicine, Nanfang Hospital, Southern Medical University, Guangzhou, China; 3 Guangzhou Center For Disease Control And Prevention (Guangzhou Health Supervision Institute), Guangzhou, China; 4 Department of clinical laboratory, Shaoxing Yuecheng People's Hospital, Shaoxing, China

**Keywords:** enterohemorrhagic *Escherichia coli*, autophagy, EspF, Annexin A6, ATG16L1

## Abstract

Autophagy is a critical host defense mechanism against pathogens; however, enterohemorrhagic *Escherichia coli* (EHEC) O157:H7 exploits it to establish infection. Here, we revealed how EHEC's effector EspF collaborates with host Annexin A6 (ANXA6) to suppress autophagy and drive inflammation. Our results showed that CRISPR/Cas9-mediated *anxa6* knockout in intestinal epithelial cells reversed EHEC-induced autophagic inhibition, as evidenced by elevated LC3B-II levels and reduced p62 accumulation. Mechanistically, EspF stabilizes ANXA6 to disrupt PI3K/mTOR signaling and impair autophagosome formation, whereas ANXA6 suppresses the expression of ATG16L1, a key autophagy regulator. In this study, EHEC infection triggered IL-1β hypersecretion in macrophages, which was coupled with NF-κB pathway hyperactivation via IκBα/p65 phosphorylation. *In vivo*, EHEC infection regulated intestinal ANXA6 expression, correlating with mucosal inflammation and barrier dysfunction. Crucially, ANXA6/ATG16L1 axis disruption created a self-reinforcing cycle of impaired autophagy, bacterial persistence, and inflammatory escalation. Our findings identified ANXA6 as a context-dependent autophagy modulator and ATG16L1 as a novel EHEC target, providing mechanistic insights into EHEC pathogenesis.

## 1. Introduction

Autophagy, a conserved lysosomal degradation pathway, serves as a frontline defense mechanism by eliminating intracellular pathogens and maintaining cellular homeostasis [1]. This catabolic process is characterized by the formation of double-membrane vesicles known as autophagosomes, which contain over 35 autophagy-related proteins (Atgs), including the autophagosome marker, microtubule-associated protein 1 light chain-3B (LC3B) [2]. These autophagosomes fuse with lysosomes to complete the degradation of the engulfed components. Host cells use this degradative process to respond to invade pathogens, and autophagy is the first line of defense [3]. Moreover, autophagy influences host immune processes, including proper immune cell differentiation, pattern recognition receptor regulation, cytokine production, inflammasome activation, antigen presentation, and lymphocyte homeostasis [4]–[6]. All these processes contribute to the host's response to pathogens.

Pathogens have evolved sophisticated countermeasures to subvert autophagic defenses. Intracellular bacteria such as Listeria monocytogenes evade autophagic capture by inhibiting Beclin1/Atg7 recruitment [7],[8], whereas Shigella flexneri blocks phagosome-lysosome fusion [9]. These strategies highlight four principal evasion paradigms: (i) Autophagy initiation suppression, (ii) autophagosome maturation impairment, (iii) autophagic recognition evasion, and (iv) the hijacking of autophagic machinery for replication [10]. However, while intracellular pathogen-autophagy interplay is well-characterized [3],[5], mechanisms employed by extracellular pathogens remain enigmatic, representing a critical knowledge gap with therapeutic implications.

Enterohemorrhagic *Escherichia coli* (EHEC) O157:H7 is an important foodborne pathogen in a group of pathogenic *E. coli* strains known as enterohemorrhagic verocytotoxin-producing organisms [8]. EHEC has a low infectious dose (50 colony-forming units) and can cause a wide range of gastrointestinal conditions, such as bloody diarrhea, hemorrhagic colitis, and life-threatening systemic hemolytic uremic syndrome (HUS) [7],[11]. The Shiga toxin produced by EHEC has been reported to induce autophagic cell death dependent on an endoplasmic reticulum stress pathway [12]. EHEC uses the T3SS-dependent effector Tir to subvert host autophagy activity in colonic epithelial cells and promote bacterial adhesion [13]. Enteropathogenic *Escherichia coli* (EPEC), which shares many virulence factors with EHEC, regulates autophagosome formation dependent on the interaction between its effector NleE and host PSMD10 protein to suppress autophagy [14]. However, a critical knowledge gap remains in our understanding of the molecular mechanisms underlying how extracellular pathogens such as EHEC exploit autophagy components to establish infection.

Central to EHEC pathogenesis is the type III secretion effector EspF, a multifunctional “molecular Swiss army knife” that disrupts mitochondrial function, triggers DNA damage, and compromises epithelial barrier integrity [15]. Our other work revealed EspF's capacity to bind structural maintenance of chromosomes 1 (SMC1) and Annexin A6 (ANXA6), inducing genomic instability and tight junction disassembly, respectively [16],[17]. ANXA6, a calcium-dependent phospholipid-binding protein, exhibits context-dependent autophagy regulation: It induces autophagic flux in cervical cancer via PI3K/AKT/mTOR inhibition [18] yet demonstrates anti-autophagic activity during viral infections [19]. This functional dichotomy raises critical questions about the role of ANXA6 in bacterial pathogenesis.

Here, building upon our discovery of EspF–ANXA6 interaction involvement in junction disruption [16], we investigate the role of these factors in autophagy and inflammatory pathogenesis. We probe the functional consequences across multiple scales, from molecular pathway analysis in intestinal epithelial cells to *in vivo* infection models. Using CRISPR/Cas9-engineered *anxa6*-knockout cells and isogenic EHEC mutants, we delineate a tripartite mechanism where: (1) EspF cooperation with ANXA6 to suppress autophagic flux via PI3K/mTOR signaling, (2) ANXA6-mediated ATG16L1 downregulation unleashes the secretion of IL-1β, and (3) this dual perturbation creates a self-reinforcing cycle of barrier dysfunction and NF-κB-driven inflammation. Our findings resolve the paradoxical anti-autophagic role of ANXA6 during bacterial infection and identify ATG16L1 as a novel host target in EHEC pathogenesis, providing mechanistic insights with therapeutic implications for enteric infections.

## 2. Materials and methods

### 2.1. Cell culture and transfection

HeLa cells stably expressing mRFP-GFP-LC3 were kindly gifted by Professor Bao Zhang (Southern Medical University, Guangzhou, China). Caco-2 cells and THP-1 cells were preserved in our laboratory. All cell lines were grown in Dulbecco's Modified Eagle Medium (DMEM, 4.5g/L glucose, Thermo Fisher Scientific, Waltham, MA, USA) enriched with 10% fetal bovine serum (FBS, ExCell Bio., Suzhou, China) and 1 × penicillin-streptomycin solution (Thermo Fisher Scientific, Waltham, MA, USA). The incubation conditions were set at 37 °C with humidified 5% CO_2_.

For cell transfection, we employed FuGENE® HD transfection reagent (Promega, Madison, WI, USA) diluted in Opti-MEM (Thermo Fisher Scientific, Waltham, MA, USA). The plasmids used (pEGFP-EspF and pEYFP-EspF-T2A-ANXA6) were prepared and stored as described [16],[20].

### 2.2. Bacterial strains and infection assay

O157:H7 EDL 933 (WT), an *espF*-deficient strain (Δ*espF*), an Δ*espF*-complementation strain (p-Δ*espF*), and DH5α were preserved in our laboratory. These strains were cultured for 16 hours at 37 °C in Luria-Bertani (LB) broth containing 0.1% kanamycin (for Δ*espF*), 0.4% chloramphenicol, and 0.1% L-arabinose (for p-Δ*espF*), as described [16]. Prior to infection, the strains were grown in DMEM (Thermo Fisher Scientific, Waltham, MA, USA) with low glucose (1 g/L) to stimulate the expression of the type three secretion system (T3SS). Cells were then exposed to the bacterial strains at a multiplicity of 100.

### 2.3. Autophagy flux assay

Tandem fluorescent-tagged LC3 (mRFP-GFP-LC3) serves as a valuable instrument for detecting autophagic flux. Upon autophagy and lysosome fusion, autolysosomes appear as red puncta, while autophagosomes are visible as yellow puncta in merged images [21]. HeLa cells stably expressing mRFP-GFP-LC3 were plated a day prior to infection or transfection. Once the cells reached 70% confluence, they were transfected with plasmids or infected with WT or Δ*espF* strains. Following a 6-hour infection or a 12-hour transfection period, the cells were washed with pre-cooled phosphate-buffered saline (PBS, Thermo Fisher Scientific, Waltham, MA, USA) and subsequently stained with DAPI (Beyotime, Shanghai, China). Intracellular autophagy was observed using an LSM880 laser scanning confocal microscope (Carl Zeiss AG, Oberkochen, Germany). The captured images were grouped, projected, and analyzed via ZEN software (Version 3.7) (Carl Zeiss AG, Oberkochen, Germany).

### 2.4. Western blotting

Cells were collected for immunoblot analysis, and protein samples were prepared accordingly. The samples were then subjected to SDS–PAGE for separation. Following electrophoresis, the proteins were transferred onto a PVDF membrane (Merck Millipore, Darmstadt, Germany). The membrane was blocked with 5% bovine serum albumin (BSA, Sigma-Aldrich, St. Louis, MO, USA) in TBST for 2 hours and subsequently incubated with a primary antibody for 16 hours at 4 °C. Afterward, the membrane was washed three times with TBST and probed with a secondary antibody. The specific antibodies employed in this investigation are enumerated in Table 1. Protein detection was facilitated using an ECL chemiluminescence substrate kit (Merck Millipore, Darmstadt, Germany). All grayscale protein analyses were performed using ImageJ software (Version 1.54) (National Institutes of Health, Bethesda, MD, USA) [22].

**Table 1.** Antibodies used in this study.

Reagent or Resource	Source	Identifiers	Dilution Ratio
monoclonal rabbit anti-LC3B	Cell Signaling Technology	3868S	1:2000
polyclonal rabbit anti-p62	Cell Signaling Technology	5114S	1:2000
monoclonal rabbit anti-ATG16L1	Cell Signaling Technology	8089S	1:2000
polyclonal rabbit anti-ANXA6	Thermo Fisher	720161	1:2000
polyclonal rabbit anti-ATG3	Cell Signaling Technology	3415S	1:2000
monoclonal rabbit anti-ATG5	Cell Signaling Technology	12994S	1:2000
monoclonal rabbit anti-ATG12	Cell Signaling Technology	4180S	1:2000
monoclonal rabbit anti-Beclin1	Cell Signaling Technology	3495S	1:2000
monoclonal rabbit anti-Akt	Cell Signaling Technology	4691S	1:2000
polyclonal rabbit anti-Phospho-Akt	Cell Signaling Technology	9271S	1:2000
monoclonal mouse anti-PI3 Kinase p85α	Proteintech	60225	1:1000
polyclonal rabbit anti-Phospho-PI3 Kinase p85α	Bioss	3332	1:1000
polyclonal rabbit anti-mTOR	Cell Signaling Technology	2972S	1:2000
polyclonal rabbit anti-Phospho-mTOR	Cell Signaling Technology	2971S	1:2000
monoclonal rabbit anti-Actin	Cell Signaling Technology	8457S	1:2000
monoclonal mouse anti-Actin	Cell Signaling Technology	3700S	1:2000
polyclonal rabbit anti-Actin	Cell Signaling Technology	4967S	1:2000
monoclonal rabbit anti-NF-κΒ p65	Cell Signaling Technology	4764S	1:1000
polyclonal rabbit anti-Phospho-NF-κB p65	Cell Signaling Technology	3031S	1:1000
monoclonal mouse anti-IκB-α	UpingBio	YP-Ab-01044	1:1000
polyclonal rabbit anti-Phospho-IκB-α	UpingBio	YP-Ab-01254	1:1000
goat anti-rabbit IgG (HRP-linked)	Cell Signaling Technology	7074S	1:4000
horse anti-mouse IgG (HRP-linked)	Cell Signaling Technology	7076S	1:4000

### 2.5. Immunofluorescence assays

Caco-2 cells were seeded into glass-bottom cell culture dishes. Once the cells reached approximately 70% confluence, they were transfected with plasmids. Forty-eight hours post-transfection, the cells underwent washing with ice-cold PBS and fixation with 4% formaldehyde for 5 minutes, followed by blocking with 10% goat serum for 1 hour. The fixed cells were then incubated with a primary antibody, either anti-LC3B (1:500, Cell Signaling Technology, Danvers, MA, USA) or anti-ANXA6 (1:500, Cell Signaling Technology, Danvers, MA, USA), diluted in 10% goat serum for 16 hours at 4 °C. Subsequently, the cells were incubated for 1 hour with a goat anti-rabbit secondary antibody (1:500, AAT Bioquest, Pleasanton, CA, USA). Next, the cells were stained with Hoechst (Beyotime, Shanghai, China) for 10 minutes at 4 °C to visualize the DNA. The prepared samples were observed using an LSM880 laser scanning confocal microscope (Carl Zeiss AG, Oberkochen, Germany), and the captured images were organized, projected, and analyzed using ZEN software (Carl Zeiss AG, Oberkochen, Germany).

### 2.6. Preparation of anxa6-knockout Caco-2 cells

CRISPR/Cas9 genome editing was employed to create *anxa6*-knockout Caco-2 cells. Lentiviral (lentiCRISPRv2) and packaging (pCD/NL-BH*DDD and pVSV-G) plasmids were sourced from Prof. Bao Zhang. The target site for *anxa6* was carefully chosen using online resources (http://crispr.mit.edu/). The designed gRNA sequences were as follows: 5′-CACCGTACTGGACATAATCACCTCAGTTT-3′ and its reverse complement 5′-AAACTGAGGTGATTATGTCCAGTACGGTG-3′. Lentivirus packaging involved co-transfecting lentiCRISPRv2-gRNA plasmids with the packaging plasmids at a 4:4:1 ratio into HEK293T cells. After 72 hours, the supernatants were collected and used to infect Caco-2 cells for an additional 72 hours, aided by 8 µg/mL polybrene (Yeasen, Shanghai, China). Stable knockout cells were then selected using 10 µg/mL puromycin (Beyotime, Shanghai, China). The knockout of *anxa6* and any off-target effects were assessed through gene sequencing, while Western blotting was used to determine the expression level of ANXA6 [23].

### 2.7. Measurement of IL-1β release by enzyme-linked immunosorbent assay (ELISA)

THP-1 cells were seeded into 6-well plates at a density of 1.2 × 10^6^ cells per well and induced to differentiate into macrophages by treatment with 100 ng/mL of phorbol myristate acetate (PMA, MedChemExpress, Monmouth Junction, NJ, USA) for 48 hours. Bacterial strains were cultured in LB broth for 16 hours at 37 °C with shaking at 220 rpm, diluted 1:50 in fresh LB broth, and grown for approximately 3 hours until the optical density at 600 nm (OD600) reached 0.5. The differentiated THP-1 cells were then infected with either O157:H7 WT or Δ*espF* strains at a multiplicity of 100 for 4 hours at 37 °C and 5% CO_2_. After infection, cell culture supernatants were collected, and interleukin-1β (IL-1β) levels were measured using the Human IL-1 beta ELISA Kit (RayBiotech, Peachtree Corners, GA, United States) following the manufacturer's instructions. Absorbance at 450 nm was determined using a Spark® multimode microplate reader (Tecan Group Ltd., Männedorf, Switzerland).

### 2.8. Measurement of lactate dehydrogenase (LDH) release

To evaluate cell injury, we quantified the release of lactate dehydrogenase (LDH) using the Cytotoxicity LDH Assay Kit from MedChemExpress (NJ, United States). We seeded Caco-2 cells, Caco-2 cells transfected with control lentiCRISPRv2 plasmids (denoted as V2), and *anxa6*-knockout Caco-2 cells (KO) into 24-well plates at a density of 2 × 10^5^ cells per well. These cells were then transfected with the plasmid pEGFP-EspF or infected with bacteria. After transfection, we collected the cell supernatants by centrifuging at 1,000 rpm for 5 minutes and transferred 50 µL to each well of a 96-well plate. Bacteria from the different groups were grown in LB broth at 37 °C and 220 rpm for 16 hours. For infection, we exposed the cells to O157:H7 wild-type (WT) or Δ*espF* strains at a multiplicity of 100 for 4 hours at 37 °C and 5% CO_2_. We then added the LDH reaction solution according to the manufacturer's instructions and measured LDH absorbance using a Spark® multimode microplate reader (Tecan Group Ltd., Männedorf, Switzerland) with a 490 nm filter. For infected cells, we used uninfected cells as the LDH-low controls. For uninfected cells, Caco-2 cells served as the LDH-low control. LDH-high controls were treated with lysis solution at 37 °C for 30 minutes. We calculated the percentage of LDH released using the formula: ([LDH sample] − [LDH low control])/([LDH high control] − [LDH low control]) × 100.

### 2.9. Immunohistochemistry

Bacteria were cultivated for 16 hours and then diluted to an approximate concentration of 5 × 10^10^ CFU/mL. Female BALB/c mice, aged 4 to 5 weeks, were randomly assigned to four groups, with eight mice per group. Each mouse received 0.2 mL of bacterial suspension (WT, Δ*espF*, p-Δ*espF*) via intragastric administration, followed by a second gavage. All mice were then intraperitoneally injected with 100 µL of mitomycin at 2.5 mg/kg to enhance infection susceptibility, as reported [24]. Mouse survival was monitored every 12 hours over a 7-day period. At the end of this period, surviving mice were euthanized by cervical dislocation, and their colon and small intestine tissues were harvested for hematoxylin and eosin (H&E) staining and immunohistochemical examination. All animal procedures were conducted in accordance with the Guide for the Care and Use of Laboratory Animals and were reviewed and approved by the Southern Medical University Experiment Animal Ethics Committee (approval number SMUL202409021).

Immunohistochemistry was conducted on 5-µm-thick sections derived from formalin-fixed, paraffin-embedded tissue blocks. The slides underwent deparaffinization and antigen retrieval with heated citric acid before being blocked with normal goat serum. The sections were subsequently incubated with primary antibodies targeting ANXA6 (1:100, Proteintech, Rosemont, IL, United States) for 16 hours at 4 °C. This step was followed by incubation with a secondary antibody (ZSGB-BIO, Beijing, China). Staining was performed using diaminobenzidine tetrahydrochloride hydrate (Sigma-Aldrich, St. Louis, MO, USA) and hematoxylin (Macklin, Beijing, China). Visualization was achieved using a NIKON digital sight DS-FI2 microscope (NIKON Corporation, Tokyo, Japan). The images were analyzed using Image-Pro Plus software (Media Cybernetics, Rockville, MD, USA), and the integrated optical density was determined for each slide.

### 2.10. Statistical analysis

All animal experiments were conducted at least twice, while *in vitro* experiments were repeated three times. The data, presented as means ± SDs, were analyzed using one-way or two-way ANOVA with SPSS software (Version 17.0) (IBM Corporation, Armonk, NY, United States). A *p* value less than 0.05 was considered to indicate significant differences between groups.

## 3. Results

### 3.1. The role of EHEC O157:H7 EspF protein and host ANXA6 protein in the autophagy process

**Figure 1.**
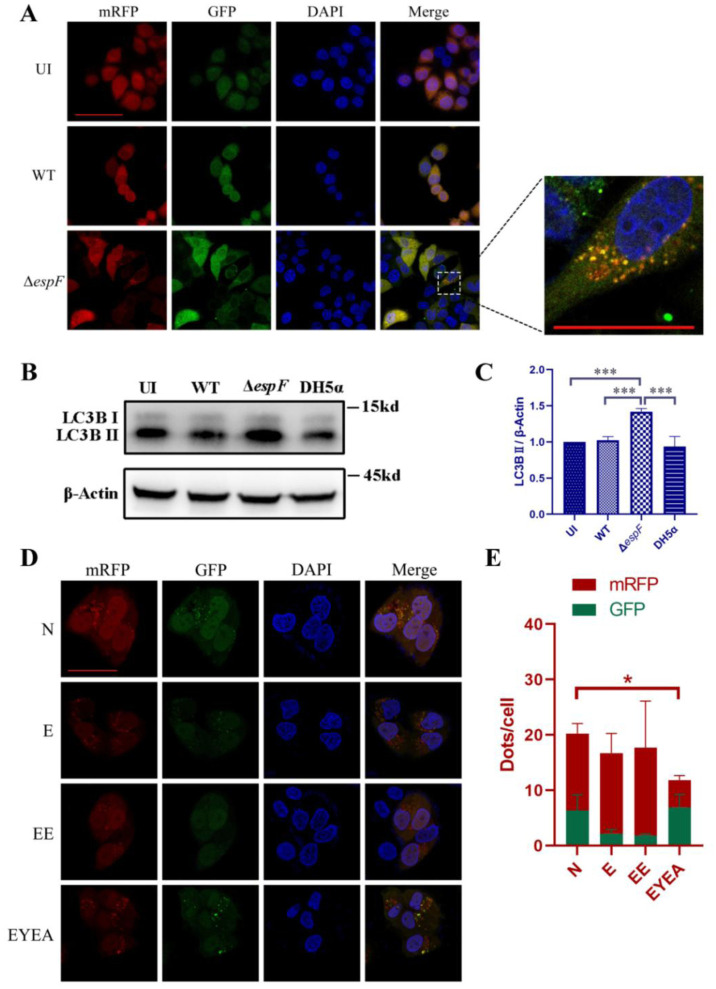
Increased LC3-positive structure number in HeLa cells subjected to *espF*-deletion O157:H7 strains and pEYFP-EspF-T2A-ANXA6 plasmids. (A) Microscopic examination of mRFP-GFP-LC3-HeLa cells six hours after infection. Immunofluorescence revealed alterations in autophagic flux, with yellow puncta inside the cells denoting autophagosomes. These images were captured using an LSM880 confocal laser scanning microscope equipped with a 63× oil objective. The scale bar represents 50 µm. (B) Expression level of LC3B in HeLa cells exposed to bacteria for six hours. (C) Band intensities of LC3B-II through densitometric measurements. The presented graph displays mean with standard deviations. UI: Uninfected HeLa cells; WT: Cells infected with wild-type O157:H7 strains; Δ*espF*: Cells infected with *espF*-deletion O157:H7 strains. DH5α: Cells infected with DH5α strains as a bacterial control. (D) Microscopic analysis of mRFP-GFP-LC3-HeLa cells transfected with plasmids. Images were acquired on an LSM880 confocal laser scanning microscope using a 63´ oil objective. The scale bar indicates a length of 50 µm. (E) Mean number of mRFP and GFP dots per cell. The results represent the means from at least three independent experiments. N: Non-transfected cells; E: Cells transfected with pEGFP; EE: Cells transfected with pEGFP-EspF; EYEA: Cells transfected with pEYFP-EspF-T2A-ANXA6; * Significant difference. * *p* < 0.05, *** *p* < 0.001.

LC3 serves as the primary molecular marker in autophagy studies [25]. We used mRFP-GFP-LC3 double-labeled HeLa cells to track autophagosome formation and breakdown, enabling us to visualize and quantify the dynamics of autophagosome formation and degradation in real-time. We infected HeLa cells with either wild-type O157:H7 (WT) or *espF*-deleted O157:H7 (Δ*espF*) strains. Immunofluorescence analysis showed that Δ*espF* strain infection notably increased LC3-positive structures, as evident in the emergence of yellow puncta representing LC3 autophagosomes (Figure 1A). This finding was paralleled by an elevation in LC3B-II levels (Figure 1B,C), indicating an EspF-specific rise in LC3-positive structures upon O157:H7 infection. As reported, EspF interacts with host ANXA6 protein, disrupting tight junctions [16],[20]. Given ANXA6's involvement in autophagy induction, we investigated the role of EspF-ANXA6 in autophagy regulation. We constructed the plasmid pEYFP-EspF-T2A-ANXA6-HA (abbreviated as pEYFP-EspF-T2A-ANXA6, EYEA), using the T2A peptide to ensure equivalent expression of *espF* and *anxa6* in the same cells, as described [16]. Notably, the EYEA group exhibited a significant reduction in LC3-positive structures compared to the control group, as revealed by immunofluorescence assay (Figure 1D,E).

**Figure 2.**
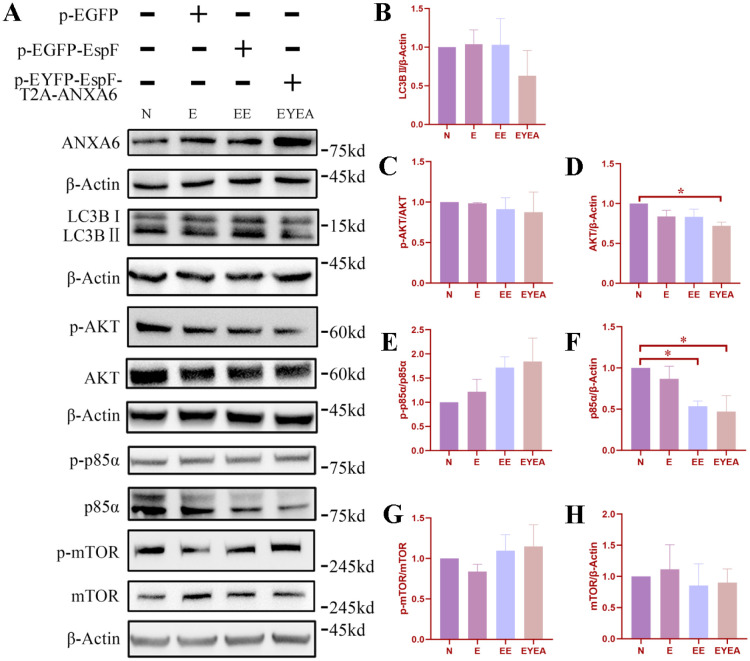
Co-expression of EspF and ANXA6 decreases the expression level of LC3B-II and may contribute to autophagy regulation via the PI3K/AKT/mTOR pathway. (A) The expression levels of ANXA6, LC3B, and key proteins involved in PI3K/AKT/mTOR signaling in HeLa cells with and without plasmid transfection. (B–H) Expression level of LC3B-II, p-AKT and AKT, p-p85α and p85α, and p-mTOR and mTOR in HeLa cells. Legends indicate non-transfected cells (N), cells transfected with pEGFP (E), cells transfected with pEGFP-EspF (EE), and cells transfected with pEYFP-EspF-T2A-ANXA6 (EYEA). * Significant difference. * *p* < 0.05.

The co-expression of EspF and ANXA6 protein (EYEA group) in HeLa cells resulted in a substantial decrease in LC3B-II expression compared to that in other groups (Figure 2A); however, the difference was not significant (Figure 2B). To explore the link between the PI3K/AKT/mTOR signaling pathway and autophagy, we assessed protein expression levels in HeLa cells transfected with various plasmids (Figure 2C–H). As observed (Figure 2A), PI3K p85α expression was significantly reduced in the EYEA and EE groups relative to the control group (Figure 2F). EspF has been reported to inhibit PI3K-dependent internalization in EPEC, and our findings further support EspF's inhibitory effect on the PI3K pathway. These results suggest that the EHEC O157:H7 EspF protein and host ANXA6 protein may be involved in autophagy via the PI3K pathway.

### 3.2. EspF upregulates ANXA6 protein expression in Caco-2 cells, and anxa6 knockout does not impact tight junction disruption by EHEC

**Figure 3.**
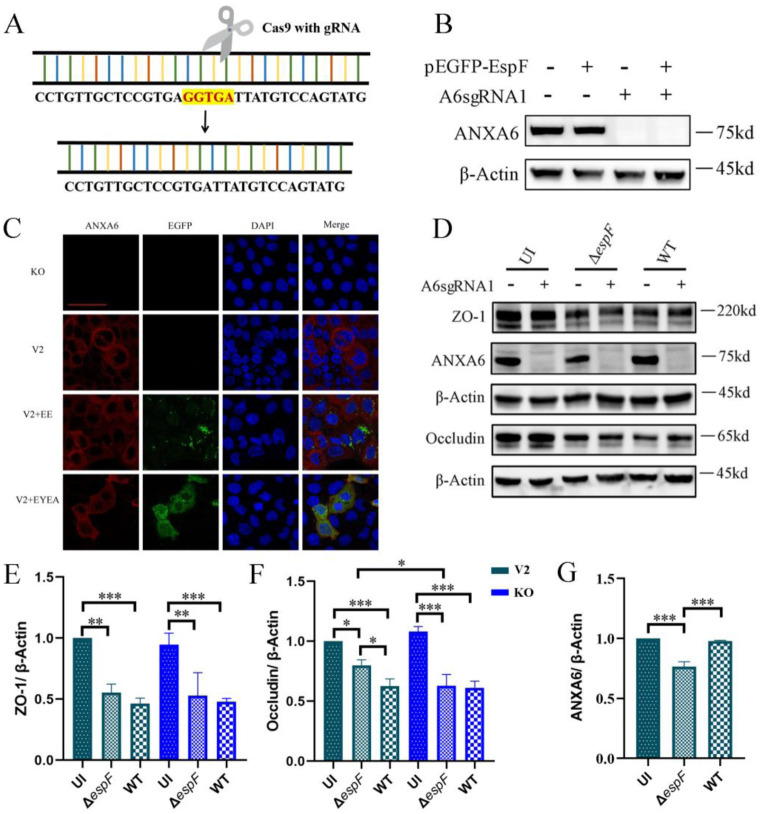
Knockout of *anxa6* in Caco-2 cells and its effects on tight junction proteins. (A) Schematic diagram highlighting the sgRNA targeting site within ANXA6. (B) Expression levels of ANXA6 in Caco-2 cells transfected with control lentiCRISPRv2 plasmids (labeled as V2) and those with lentiCRISPRv2-A6sgRNA1 plasmids (denoted as KO). (C) Immunofluorescence microscopy images of ANXA6, with a scale bar indicating 50 µm. KO: Caco-2 cells with *anxa6* knockout. V2+EE: V2 cells transfected with plasmid pEGFP-EspF; V2+EYEA: V2 cells transfected with pEYFP-EspF-T2A-ANXA6 plasmids. The nucleus is stained blue with Hoechst, ANXA6 appears red due to anti-ANXA6 antibody labeling, and green signifies fluorescence emitted by the fluorescent plasmid. These images were captured using an LSM880 confocal laser scanning microscope equipped with a 63´ oil objective. (D) Expression levels of ANXA6, ZO-1, and occludin in V2 and KO cells exposed to bacteria for 6 hours. (E–G) Quantitative analysis of band intensities of ZO-1, occludin, and ANXA6 through densitometric measurements. * Significant difference. * *p* < 0.05, ** *p* < 0.01, *** *p* < 0.001.

Given that EHEC predominantly targets human intestinal epithelial cells and that Caco-2 cells are the optimal model for simulating a monolayer of intestinal epithelial cells [26], we chose the Caco-2 cell line for our studies. Furthermore, our prior research has demonstrated that EspF interacts with host ANXA6, leading to the disruption of host tight junctions [16]. This finding suggests a potential role for ANXA6 in EHEC pathogenesis. To further investigate the function of ANXA6, we employed CRISPR-Cas9 to knock out ANXA6 expression in Caco-2 cells using sgRNA targeting *anxa6* (Figure 3A). Our findings revealed minimal or undetectable ANXA6 expressions in Caco-2 cells with A6sgRNA (labeled KO), contrasting sharply with control cells (labeled V2) (Figure 3B). Immunofluorescence assays further confirmed ANXA6's primary localization to the cell membrane and cytoplasm in Caco-2 cells, with virtually no expression in KO cells (Figure 3C). We then assessed the expression levels of ANXA6 and tight junction proteins in V2 and KO cells upon EHEC infection. Notably, ANXA6 expression was significantly reduced in the Δ*espF* group compared to that in the WT group (refer to Figure 3D,G). However, ANXA6 expression levels in the WT and uninfected groups were comparable. Then we detected the background expression of ANXA6 in Caco-2 tumor cells and observed significantly higher ANXA6 expression compared to human intestinal epithelial cells (HIEC) (data not shown). This inherently high basal expression in Caco-2 cells may mask potential differences between the WT-infected and uninfected groups, thereby limiting our ability to detect infection-dependent changes in this cell line. The expression of ZO-1 and Occludin remained largely unchanged in V2 and KO cells, but decreased significantly upon infection with WT or Δ*espF* strains (Figure 3D–F). These findings imply that knockout of *anxa6* does not affect intestinal tight junction structures or alter the downregulation of tight junctions induced by EHEC infection.

### 3.3. EspF-ANXA6 inhibited autophagy, whereas anxa6 knocked out enhanced autophagy

**Figure 4.**
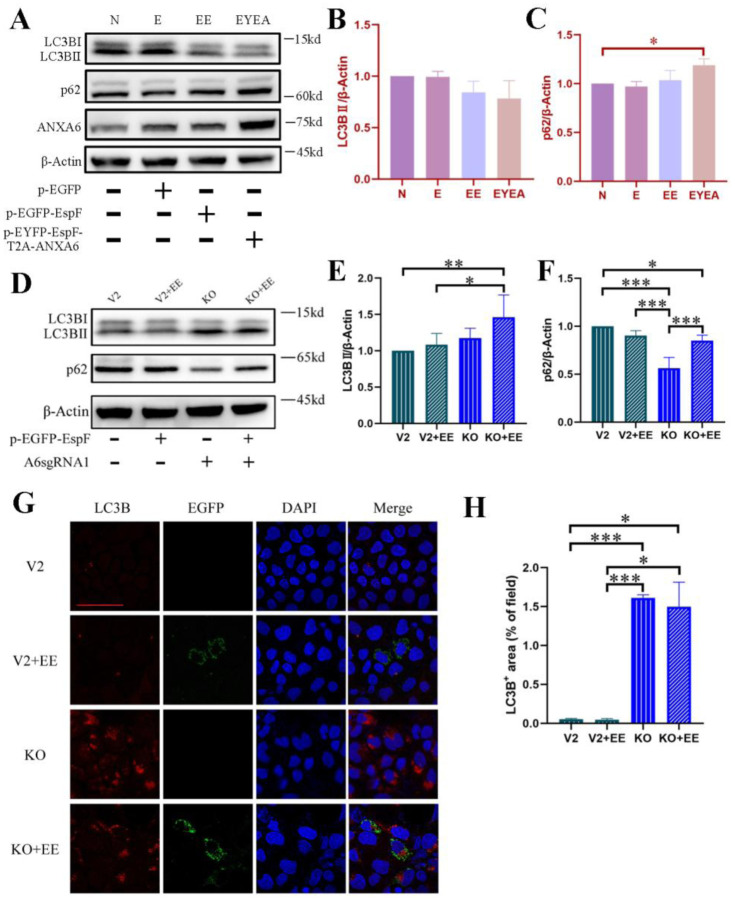
The role of EspF and ANXA6 in autophagy regulation. (A) Expression level of ANXA6, LC3B, and p62 in Caco-2 cells with and without plasmid transfection. (B,C) Band intensities of LC3B-II and p62 in Caco-2 cells were measured densitometrically. N: Non-transfected cells; E: Cells transfected with pEGFP; EE: Cells transfected with pEGFP-EspF; EYEA: Cells transfected with pEYFP-EspF-T2A-ANXA6. (D) Expression level of LC3B and p62 in Caco-2 cells with and without *anxa6* knockout. (E,F) Band intensities of LC3B-II and p62 were measured densitometrically. (G) Immunofluorescence microscopy of LC3B captured using an LSM880 confocal laser scanning microscope with a 63´ oil objective. The scale bar denotes 50 µm. (H) Image intensities of LC3B. The results represent the means from at least three independent experiments. V2: Caco-2 cells transfected with control lentiCRISPRv2 plasmids. KO: Caco-2 cells with *anxa6* knockout. V2+EE: V2 cells transfected with pEGFP-EspF. KO+EE: KO cells transfected with pEGFP-EspF. * Significant difference. * *p* < 0.05.

We conducted further investigations to elucidate the role of EspF-ANXA6 in modulating autophagy within Caco-2 cells. The expression level of LC3B-II remained relatively consistent across groups (Figure 4A), with the EYEA group showing a marginal decrease compared to the control group (N), although the difference was not significant (Figure 4B). The autophagy substrate protein p62 demonstrated a notable increase in the EYEA group relative to the control group (Figure 4C). Our exploration of ANXA6's function in autophagy regulation revealed that genetically knocking out *anxa6* led to a significant boost in autophagy activity within Caco-2 cells. This enhancement was evidenced by a marked elevation in LC3B-II expression and accumulation of LC3B puncta in KO cells and those transfected with pEGFP-EspF (KO+EE) (Figure 4D–E,G and H). Moreover, the autophagy substrate protein p62 was substantially reduced in the knockout groups relative to that in the control (as depicted in Figure 4D,F). Interestingly, p62 levels were higher in the KO+EE group than those in the KO group, indicating EspF's potential involvement in autophagy modulation. These results demonstrate that ANXA6 plays a critical role in suppressing autophagy, and that EspF modulates autophagy in an ANXA6-dependent manner. The lack of further change in LC3B-II levels upon EspF expression in *anxa6*-knockout cells indicates that EspF requires ANXA6 to exert its regulatory function.

### 3.4. EspF-ANXA6 hinders autophagy by modifying the process of autophagic membrane formation and regulating the PI3K/mTOR pathway

**Figure 5.**
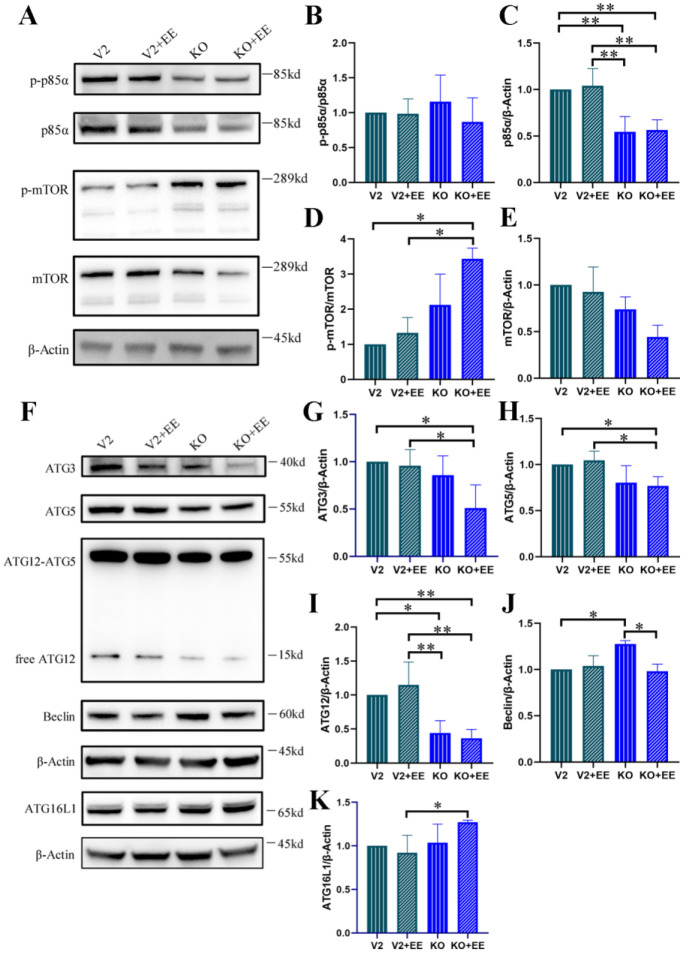
EspF-ANXA6 suppresses autophagy by altering the autophagic membrane formation process and regulating the PI3K/mTOR pathway. (A) Expression level of PI3K/mTOR signaling proteins. (B–E) Band intensities of PI3K/mTOR signaling proteins were measured densitometrically. V2: Caco-2 cells transfected with control lentiCRISPRv2 plasmids. KO: Caco-2 cells with *anxa6* knockout. V2+EE: V2 cells transfected with pEGFP-EspF. KO+EE: KO cells transfected with pEGFP-EspF. (F) Expression level of autophagy-related proteins: ATG3, ATG5, ATG12, Beclin, and ATG16L1. (G–K) Band intensities of autophagy-related proteins were measured densitometrically. * Significant difference. * *p* < 0.05, ** *p* < 0.01, *** *p* < 0.001.

Since ANXA6 is known to negatively regulate autophagy, we further investigated how EspF-ANXA6 affects the PI3K/mTOR signaling pathway in Caco-2 cells with and without *anxa6* knockout. PI3K p85α and its phosphorylated form (p-p85α) were notably reduced after *anxa6* knockout (Figure 5A–C), indicating that ANXA6 might influence PI3K signaling. Furthermore, in the KO+EE group, *anxa6* knockout in combination with EspF expression resulted in elevated mTOR phosphorylation (p-mTOR) despite a modest reduction in total mTOR protein, indicating that pathway activity is not solely determined by mTOR abundance (Figur6e 5D,E). In contrast, as noted, neither mTOR nor p-mTOR expression showed substantial changes across all experimental groups in HeLa cells (Figure 2G,H). We attribute this discrepancy to inherent differences between the Caco-2 and HeLa cell lines. Moreover, the results in Caco-2 cells suggest that the ANXA6 protein regulates mTOR activity primarily by modulating mTOR phosphorylation status rather than altering total mTOR protein levels during autophagy.

In Caco-2 cells, *anxa6* knockout combined with EspF expression led to increased mTOR phosphorylation alongside a reduction in total mTOR protein, suggesting complex post-translational regulation. However, autophagy modulation in this system is primarily governed by the activation state of the PI3K/AKT/mTOR pathway, as inferred from phosphorylation dynamics and downstream autophagic flux.

Studies have shown that ANXA6 functions in vesicle fusion by binding to negatively charged phospholipids on membranes [27],[28]. ATG3, an E2-like enzyme, interacts with ATG7 and facilitates the conjugation of ATG12, a critical step in autophagy leading to the formation of the ATG12-ATG5 conjugate and subsequent autophagosomal membrane formation [29]. To understand how EspF-ANXA6 inhibits autophagy, we examined the expression of proteins involved in autophagosome membrane formation. In KO cells, we observed a significant downregulation of ATG3, ATG5, and ATG12, which are essential for LC3 lipidation and autophagosome formation (Figure 5F–I). The expression of Beclin1, a central player in autophagy that interacts with multiple factors to mediate autophagy protein localization and promote autophagy precursor structure formation, was significantly upregulated in the knockout group compared to that in the control and KO+EE groups (Figure 5J). Notably, *anxa6* knockout significantly increased ATG16L1 protein expression in KO+EE group (Figure 5K). ATG16L1 is a vital component of the autophagic machinery that modulates the endotoxin-induced intestinal inflammatory response; under certain conditions, autophagy can influence IL-1β processing and secretion [30], but its primary homeostatic role is to restrain excessive inflammasome activation. These data suggest that EspF-ANXA6 regulates autophagy by altering the autophagic membrane formation process, and ATG16L1 might be a potential target in EHEC pathogenesis.

### 3.5. Inflammatory responses during EHEC infection depend on the EspF-ANXA6 complex and ATG16L1 downregulation

**Figure 6.**
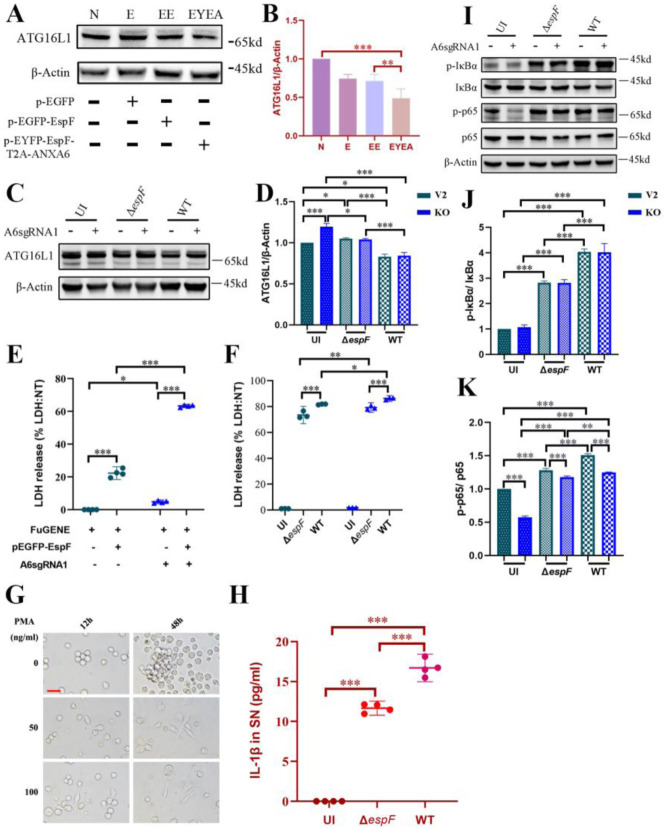
EspF-ANXA6 interaction leads to a decrease in ATG16L1 levels, potentially contributing to inflammation triggered by O157:H7 infection. Expression level and band intensities of ATG16L1 in Caco-2 cells transfected with various plasmids (A,B) or infected with different bacterial strains (C,D). N: Non-transfected cells; E: Cells transfected with control plasmid pEGFP; EE: Cells transfected with plasmid pEGFP-EspF; EYEA: Cells transfected with plasmid pEYFP-EspF-T2A-ANXA6. (E,F) Percentage of released LDH in V2 and KO cells under plasmid transfection or bacterial infection conditions. (G) THP-1 cells were differentiated with PMA at 100 ng/mL. Images were taken by NIKON NIS-Elements Ti2-U microscope at 400× magnification. The scale bar represents 50 µm. (H) Release of IL-1β in THP-1 cells infected with bacteria. (I) Expression level of NF-κB signaling pathway proteins in Caco-2 (V2 and KO) cells infected with bacteria. (J,K) Band intensities of p65 and IκBα were measured densitometrically. V2: Caco-2 cells transfected with control lentiCRISPRv2 plasmids. KO: Caco-2 cells with *anxa6* knockout. V2+EE: V2 cells transfected with pEGFP-EspF. KO+EE: KO cells transfected with pEGFP-EspF. UI: Uninfected HeLa cells; WT: Cells infected with wild-type O157:H7 strains; Δ*espF*: Cells infected with *espF*-deletion O157:H7 strains. * Significant difference. * *p* < 0.05, ** *p* < 0.01, *** *p* < 0.001.

Our study emphasized the significant role of the EspF-ANXA6 complex in shaping inflammatory reactions during EHEC infection. When EspF and ANXA6 were co-expressed in Caco-2 cells, ATG16L1 expression notably decreased (Figure 6A,B). In V2 and KO cells, ATG16L1 expression was lower in the WT group compared to the uninfected controls, whereas infection with *espF*-deletion strains reduced this decline (Figure 6C,D). Aligning with earlier findings (see Figure 5F), knockout of *anxa6* in uninfected cells led to elevated ATG16L1 expression. EHEC infection downregulates ATG16L1 in an EspF-dependent manner. While this effect occurs in both ANXA6-sufficient and -deficient cells, the magnitude of suppression is greater when ANXA6 is present, suggesting that ANXA6 acts as a co-factor that amplifies EspF-mediated inhibition of ATG16L1. ATG16L1 is crucial in regulating inflammatory responses and autoimmune diseases by suppressing IL-1β signaling [30], and its inhibition by EHEC may trigger host inflammatory reactions. However, we were unable to detect IL-1β in either V2 or KO cells (data not shown). Additionally, Caco-2 cells with *anxa6* knockout and transfected with pEGFP-EspF showed a trend toward increased LDH release compared to V2 cells transfected with pEGFP-EspF alone (Figure 6E), suggesting a potential additive effect of ANXA6 deficiency and EspF expression on cellular damage. However, the absolute increase was modest, and both conditions contributed to elevated LDH levels relative to non-transfected controls. Infection with WT EHEC resulted in higher LDH release than Δ*espF* infection (Figure 6F), consistent with a role for EspF in promoting cell injury. However, the difference between KO and V2 cells was relatively small, and both exhibited substantial LDH release, indicating that multiple factors contribute to epithelial barrier disruption during infection.

Since Caco-2 cells are not immune cells, we subsequently assessed IL-1β levels in THP-1 cells. We induced THP-1 cells to differentiate into macrophages using PMA at 100 ng/mL (Figure 6G). O157:H7 infection elevated IL-1β release in THP-1 cells; however, this effect was reduced when *espF* was deleted from the O157:H7 strains (Figure 6H). We also examined the classical NF-κB signaling pathway during O157:H7 infection in Caco-2 cells. Key factors of this pathway, IκBα and p65, underwent phosphorylation in both WT and Δ*espF* groups; however, phosphorylation levels decreased when *espF* was deleted (Figure 6I–K). The inflammatory responses observed during O157:H7 infection, evidenced by LDH release, IL-1β production, and NF-κB activation, are partially dependent on EspF and amplified through its interaction with ANXA6 and subsequent downregulation of ATG16L1, although other EHEC virulence factors undoubtedly contribute to the overall pathogenic outcome.

### 3.6. ANXA6 expression increased in mice infected with O157:H7, suggesting its potential involvement in intestinal mucosal inflammation

**Figure 7.**
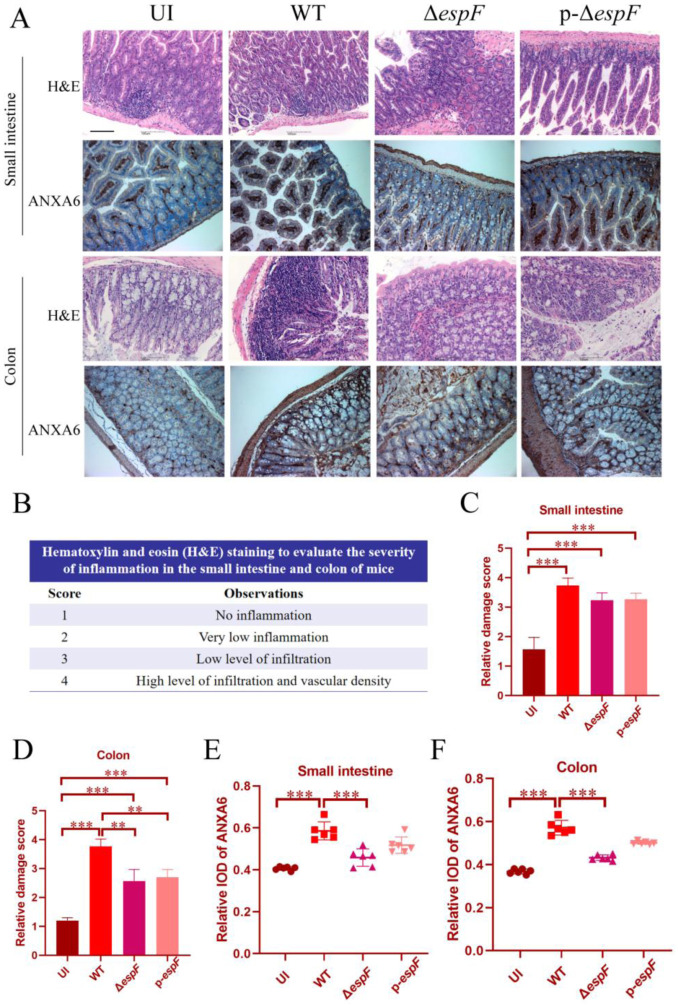
Hematoxylin and eosin (H&E) staining and immunohistochemistry of ANXA6 in BALB/c mice infected with O157:H7 strains. (A) Infection with O157:H7 induces lesions in the small intestine and colon, an effect mitigated by the Δ*espF* strain. Notably, ANXA6 expression increased upon O157:H7 infection. Images were captured using a NIKON digital sight DS-FI2 microscope at 200´ magnification; scale bar represents 100 µm. (B–D) H&E staining reveals the severity of inflammation in the mouse small intestine and colon, quantified using a relative damage score. (E,F) Integrated option density (IOD) analysis quantifying ANXA6 levels in the small intestine and colon. Displayed data are mean ± SD. UI: uninfected. WT: BALB/c mice infected with wild-type O157:H7 strain. Δ*espF*: BALB/c mice infected with *espF*-deficient O157:H7 strain, p-Δ*espF*: BALB/c mice infected with *espF*-complementation strain. * Significant difference. ** *p* < 0.01. *** *p* < 0.001.

To further investigate the role of ANXA6 during O157:H7 intestinal infection, we exposed mice to O157:H7 wild-type (WT), *espF*-deficient (Δ*espF*), and *espF*-complemented (p-Δ*espF*) strains. We evaluated the intestinal mucosal barrier's integrity through H&E staining and measured ANXA6 expression in the small intestine and colon. Histological analysis showed lymphocytic infiltrates in the WT group's small intestine and colon; these infiltrates were reduced upon *espF* deletion (Figure 7A–D). Additionally, compared to the control and Δ*espF* groups, ANXA6 protein levels were notably higher in the WT group's small intestine and colon (Figure 7E,F). These results emphasize the significance of ANXA6 in the inflammatory response of the intestinal mucosa triggered by O157:H7 infection.

## 4. Discussion

Autophagy, a conserved cellular degradation pathway essential for maintaining homeostasis through organelle and protein recycling [31],[32], plays a pivotal role in host defense against intracellular pathogens [33]. While numerous bacteria have evolved mechanisms to subvert this process [34],[35], the exploitation of autophagy by extracellular pathogens for adhesion and survival remains unclear. Our study reveals a novel mechanism by which EHEC O157:H7 targets host ANXA6 and ATG16L1, thereby suppressing autophagic flux and exacerbating inflammatory responses. These findings enhance our understanding of how extracellular pathogens manipulate cellular homeostasis to establish infection and provoke disease.

Investigations have established that O157:H7 Shiga toxins induce autophagic cell death through ER stress pathways [12],[36], while the Tir effector activates PKA to inhibit autophagy and promote epithelial colonization [13]. Building on our prior discovery that EHEC's EspF effector disrupts tight junctions via ANXA6-mediated MLCK signaling [16], we demonstrate that EspF-ANXA6 interaction constitutes a multimodal virulence mechanism. In cells expressing both EspF and ANXA6, the reduction in LC3B-II levels and accumulation of p62 indicate a blockade in autophagosome maturation or lysosomal degradation, similar to Listeria monocytogenes' strategies to block autophagosome-lysosome fusion [7], yet distinct in its exploitation of ANXA6. ANXA6 is a key negative regulator of autophagy in Caco-2 cells. EspF modulates autophagy primarily through interaction with ANXA6, and its effects are dependent on ANXA6 expression. When ANXA6 is absent, EspF fails to fully suppress autophagy, suggesting that EspF requires ANXA6 as a functional partner to inhibit autophagic flux. Therefore, the EspF-ANXA6 axis acts as a critical regulator of autophagy during EHEC infection, but EspF alone is insufficient to regulate autophagy in the absence of ANXA6. This calcium-dependent phospholipid-binding protein, characterized as an autophagy inducer in cancer models [18],[37], exhibits paradoxical anti-autophagic activity during EHEC infection. We propose this functional dichotomy arises from pathogen-induced post-translational modifications or context-dependent rewiring of stress response pathways, exemplifying the evolutionary adaptability of bacterial effectors in repurposing host factors.

In mouse experiments, we observed that EHEC infection upregulates ANXA6 protein expression, whereas *espF* gene deletion attenuates this effect. The upregulation of ANXA6 expression in EHEC-infected murine intestines and its association with mucosal inflammation suggest ANXA6 as a potential biomarker and therapeutic target. ANXA6 protein has also been reported to be enriched in fecal samples from patients with colorectal cancer [38],[39]. We further discovered that ANXA6 protein expression is significantly higher in Caco-2 cells compared to that in intestinal epithelial HIEC cells, which may contribute to its role in inhibiting autophagy. While ANXA6's role in membrane repair and exocytosis [40] may initially mitigate epithelial damage, we speculate that sustained expression during infection likely perpetuates inflammation by maintaining EspF activity. However, this requires further investigation.

A notable decrease in ATG16L1 levels was observed in Caco-2 cells expressing both EspF and ANXA6, indicating that the EspF-ANXA6 complex suppresses ATG16L1 expression. Furthermore, genetic ablation of ANXA6 led to increased ATG16L1 expression, suggesting that ANXA6 negatively regulates ATG16L1. Mechanistically, ATG16L1 deficiency disrupts intestinal homeostasis through two convergent pathways: First, by failing to suppress NLRP3 inflammasome activation and subsequent IL-1β overproduction [30]; and second, by amplifying proinflammatory signals downstream of bacterial pattern recognition receptors NOD1 and NOD2 [41]. Our observations of elevated IL-1β secretion in WT EHEC-infected macrophages, coupled with enhanced phosphorylation of IκBα and NF-κB p65, directly link EspF-ANXA6-mediated ATG16L1 suppression to NF-κB pathway hyperactivation.

The clinical relevance of this mechanism is underscored by human genetic studies associating ATG16L1 polymorphisms (e.g., T300A) with exaggerated IL-1β and IL-6 production in inflammatory bowel disease [30],[42],[43]. Importantly, ATG16L1's anti-inflammatory functions are mechanistically intertwined with its autophagy-promoting activity. By sustaining autophagic clearance of damaged organelles and protein aggregates, ATG16L1 prevents epithelial cell death and preserves intestinal barrier integrity [44]. The coordinated downregulation of ATG16L1 and autophagy impairment induced by EspF-ANXA6 thus creates a self-reinforcing pathogenic cycle: Compromised barrier function facilitates bacterial persistence, which in turn exacerbates inflammation through unrestrained NF-κB activation and inflammasome signaling. This tripartite interaction positions the ANXA6/ATG16L1 axis as a critical molecular hub connecting EHEC infection, autophagy dysfunction, and inflammatory tissue damage in the gut.

Although we outline the EspF-ANXA6-ATG16L1 axis, several points remain. First, although the LC3B-II/p62 flux analysis suggests autophagic blockade, incorporating pharmacological inhibitors (e.g., chloroquine or bafilomycin) would strengthen the mechanistic interpretation. Second, proteomic and epigenetic analyses are needed to determine whether the precise mode of ATG16L1 suppression is transcriptional (via promoter methylation), translational (miRNA-mediated), or post-translational (ubiquitination). Third, *in vivo* studies utilizing conditional *anxa6*-knockout mice are needed to clarify cell-type-specific effects in the intestinal epithelium versus immune cells. Fourth, our work relies on EHEC strains expressing Stx, and we acknowledge that the contribution of EspF-ANXA6 signaling independent of Stx cannot be fully disentangled; future studies employing isogenic Stx-deficient (Δ*stx*) EHEC strains would be valuable to isolate the toxin-independent roles of this pathway in inflammation and autophagy. Finally, the preclinical testing of therapeutic strategies targeting EspF-ANXA6 interactions or enhancing ATG16L1 function is warranted.

**Figure 8.**
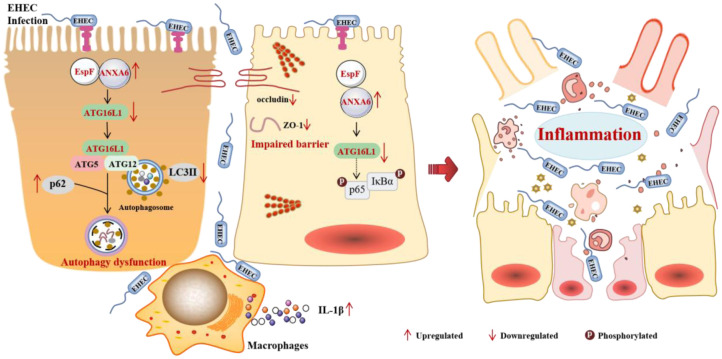
An integrative model of EHEC infection, inhibition of autophagy via an EspF-ANXA6-dependent mechanism, and suppression of ATG16L1 to induce inflammation. EspF interacts with the host ANXA6 protein, leading to a downregulation of LC3B-II expression and an increase in p62 protein levels, thereby inhibiting autophagy. Furthermore, the interaction between EspF and ANXA6 results in the downregulation of the ATG16L1 protein. EHEC may disrupt the intestinal barrier via ATG16L1, enabling its entry into the host and subsequent activation of the NF-κB signaling pathway, which promotes the release of IL-1β. These effects contribute to the onset of inflammation.

In conclusion, we elucidate a tripartite mechanism wherein EHEC's EspF effector coopts ANXA6 to suppress ATG16L1-dependent autophagy, resulting in NF-κB-mediated inflammation (Figure 8). These findings expand our understanding of bacterial autophagy evasion tactics and highlight ANXA6 and ATG16L1 as potential targets for addressing EHEC-induced pathologies. Our findings indicate potential novel therapeutic targets for curbing EHEC pathogenesis and underscore the intricate interplay among membrane dynamics, autophagy, and inflammation.

## Use of AI tools declaration

The authors declare they have not used Artificial Intelligence (AI) tools in the creation of this article.
